# P-885. Clinical Characteristics and Molecular Epidemiology of Invasive Nontypeable *Haemophilus influenzae* Disease at a Tertiary Medical Center in Washington, DC

**DOI:** 10.1093/ofid/ofae631.1076

**Published:** 2025-01-29

**Authors:** Stephen Stone, Matthew Spence, Afsoon Roberts, Maria Elena Ruiz, Marc O Siegel, Lauren F Collins, Sarah Lohsen, Sarah W Satola, Rebecca Yee, Jose A Lucar

**Affiliations:** George Washington University, Falls Church, Virginia; George Washington University Hospital, Washington, District of Columbia; George Washington University, Falls Church, Virginia; GWU, Washington DC, District of Columbia; George Washington University School of Medicine and Health Sciences, Washington, District of Columbia; Emory University School of Medicine, Division of Infectious Diseases, Atlanta, Georgia; Emory University School of Medicine, Atlanta, Georgia; Emory University School of Medicine, Division of Infectious Diseases, Atlanta, Georgia; George Washington University School of Medicine and Health Sciences, Washington, District of Columbia; George Washington University, Falls Church, Virginia

## Abstract

**Background:**

The incidence of invasive nontypeable H. influenzae (NTHi) is on the rise, and NTHi now causes the majority of invasive H. influenzae infections across all age groups. While these presentations are usually among people at the extremes of age, we observed a recent increase in invasive NTHi cases in adults at our institution compared to previous years. Here, we report their clinical presentation and the subsequent molecular epidemiology investigation.

Characteristics of Patients With Invasive Nontypeable Haemophilus influenzae (NTHi) Infection, GW Hospital, December 2022-August 2023

**Figure:**
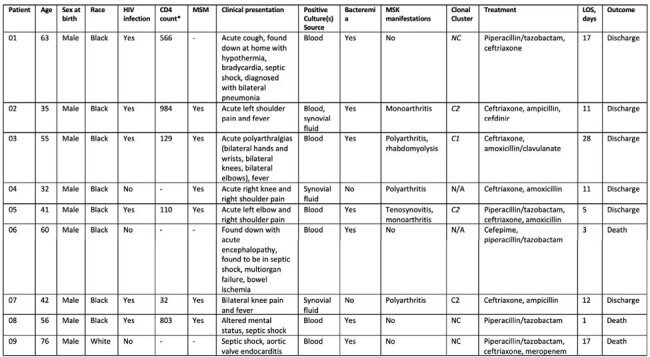

Abbreviations: HIV, human immunodeficiency virus; MSM, men who have sex with men; MSK, musculoskeletal; LOS, length of hospital stay; N/A, information not available; HBV, hepatitis B virus; C1, clonal cluster 1; C2 clonal cluster 2; NC, non-cluster

* in cells/mm3

**Methods:**

We describe the demographics, clinical characteristics, and outcomes of individuals hospitalized at our institution with invasive NTHi disease between December 2022 and August 2023. Invasive NTHi disease was defined as infections beyond the respiratory tract or skin. Data was obtained from retrospective chart abstraction. Available bacterial isolates were sent to the Georgia Emerging Infections Program laboratory for polymerase chain reaction (PCR) and whole genome sequencing (WGS), and were categorized as belonging to clusters 1 or 2 (C1, C2) or as non-cluster, as described previously.

**Results:**

Nine cases [median age 55 (32-76) years, 100% male, 89% Black] were identified. Five individuals were living with HIV (PWH) on antiretroviral therapy, with median CD4 count 348 (range 32-984) cells/mm^3^, and all except one were virologically suppressed. Six patients identified as men who have sex with men (MSM). Five patients presented with musculoskeletal manifestations. Seven isolates were available for PCR/WGS: one isolate belonged to C1, three to C2, and three as non-cluster. The mean hospital stay was 11 (range 3-28) days. Three patients required admission to the intensive care unit, all of which died.

**Conclusion:**

This case series identified nine patients within a nine-month span presenting with invasive NTHi disease, an abrupt rise from our local historical data. We noted an overrepresentation of PWH and MSM, with over half of isolates belonging to two previously identified clonal clusters associated with similar demographics. Future research is needed to better understand novel transmission routes, clinical characteristics, genotypic relatedness, and geographic spread of invasive NTHi disease.

**Disclosures:**

**Rebecca Yee, Ph.D**, Biomerieux: Grant/Research Support

